# Vaccination With *Trichuris muris* Poly‐Cysteine and Histidine‐Tailed Protein p43 Protects Against Chronic Infection

**DOI:** 10.1111/pim.70107

**Published:** 2026-09-30

**Authors:** Allison J. Bancroft, Kelly S. Hayes, Laura Campbell, Seona Thompson, Christina Dold, Luke Blackwell, Christine S. Rollier, David J. Thornton, Richard K. Grencis

**Affiliations:** ^1^ Lydia Becker Institute for Immunology and Inflammation Manchester UK; ^2^ Manchester Cell Matrix Centre Manchester UK; ^3^ Division of Infection, Immunity and Respiratory Medicine University of Manchester Manchester UK; ^4^ Faculty of Biology, Medicine and Health, School of Biological Sciences, Manchester Academic Health Science Centre University of Manchester Manchester UK; ^5^ Department of Paediatrics Oxford Vaccine Group, University of Oxford and the NIHR Oxford Biomedical Research Centre, CCVTM, Churchill Lane Oxford UK

**Keywords:** chronic infection, immunity, nematode, Type 2 immunity, vaccination, whipworm

## Abstract

*Trichuris trichiura* is one of the soil transmitted geohelminths which together currently affect approximately 25% of the global population resulting in considerable morbidity particularly in children and young adults. Recent estimates put infection by *T. trichiura* at approximately 513 million. De‐worming programmes are often not completely effective against *T. trichiura* and fail to provide resistance to reinfection. Vaccination against these multicellular parasites has proved difficult as they have complex antigenic make‐up and the potential to harness the host immune system to their own advantage. Previous vaccination experiments in mice using excretory secretory (ES) material have proved successful although the specific antigens responsible for protection have remained elusive. Here we show that vaccination of mice with native (n)p43, the dominant secreted protein from adult *T. muris*, formulated with Alum adjuvant can protect against chronic infection. The vaccine candidate generated a strong Type 2 response and host protection was dependent on signalling through IL‐4 Rα. Vaccination with a recombinant (r)p43 formulated with alum resulted in stunted worms and reduced worm fecundity but no significant reduction in worm burden. Deglycosylation of np43 significantly abrogated protection against infection suggesting p43 glycans play a role in mediating the protective efficacy of np43. Vaccination using an adenoviral p43 construct generated a robust antibody response to np43 but no protection against challenge infection. Orthologues of p43 are present in other closely related nematodes including *T. trichiura and T. suis* which infects wild and domestic pigs, suggesting potential for similar vaccine development for both human and veterinary trichiuriasis.

## 1 Introduction

The soil transmitted helminths (STH), namely *Ascaris lumbricoides*, *Trichuris trichiura*, and the hookworms, *Ancylostoma duodenale* and *Necator americanus* , have considerable global prevalence, with 1/17 people worldwide being infected with whipworm, *T. trichiura* [1, 2]. Although rarely fatal, whipworms cause considerable morbidity, affect economic growth in some of the poorest areas of the world, and result in 4.5–39 million Disability Adjusted Life Years (DALYs) per annum. Children are most affected, resulting in stunted growth, cognitive impairment [3] and finger clubbing. Adults and children can also suffer from Trichuris dysentery syndrome and colitis [4, 5]. Furthermore, current anthelmintics (albendazole and mebendazole) are not completely effective against *Trichuris*, and individuals become rapidly re‐infected after deworming [6]. These anthelmintics are also associated with increasing drug resistance [7]. A recent study predicted the speed of emerging resistance to preventative drug treatment and suggested this could appear within the next decade [8]. Moreover, recent studies have identified a new species that infects primates and humans, *T. incognita*, that exhibits significant resistance to conventional anthelmintic treatment [9, 10]. Thererfore, development of a safe and effective vaccine would be a major advance, however, studies to develop a pan helminth vaccine have so far proved unsuccessful [11, 12].

The natural mouse parasite *Trichuris muris* has been used for many years as a model for *T. trichiura* to investigate the protective host immunity including post vaccination. The majority of vaccination studies have used excretory/secretory products from adult worms which comprise a complex mixture of hundreds of proteins [13, 14, 15]. Moreover, in most vaccination studies, animals were challenged with a high infection dose, which the majority of inbred mouse strains naturally expel from the intestine via a Type 2 response, so vaccine efficacy was assessed by an acceleration of the “normal” rate of worm expulsion [15, 16, 17]. Natural infection by most STH, including *T. muris*, is through low numbers of infectious stages. A low dose infection of *T. muris* eggs is not expelled from the intestine resulting in a long‐lived patent infection, reflective of natural *T. muris* infection and other STH infections, that is chronic infection with low numbers of parasites [18]. Furthermore, a low dose *T. muris* infection does not drive a host protective Type 2 response but induces a Type 1 response which underpins susceptibility [19]. Indeed, induction of strong host protective Type 2 immunity following low dose infection is difficult to achieve [20]. Thus, the low dose challenge infection is a rigorous and robust model for assessing vaccine efficacy.

We have previously shown that immunisation with Excretory Secretory (ES) products from adult *T. muris*, which contain a mixture of hundreds of potential protective antigens, can protect against a subsequent low dose challenge infection although the precise antigens responsible for this are still unknown [13]. Within the complex mixture of adult *T. muris* ES, a single protein dominates in terms of quantity, p43. We solved the crystal structure of p43 and showed it is a secreted immunomodulatory molecule that binds to extracellular matrix glycosaminoglycans and can block the function of the host Type 2 cytokine interleukin (IL) ‐13, which is critical for expulsion of the parasite from the intestine [21]. Remarkably, we also showed that p43 did not induce a detectable antibody or T cell response following natural *T. muris* infection while immunisation with purified p43 and alum adjuvant generated a robust adaptive immune response and resulted in protection against a low dose challenge infection [13, 21].

Here, we extend our previous studies by investigating the nature of the vaccine immune response required for protection, the vaccination potential of both recombinant p43 and an adenoviral delivered p43 vaccine, and the role of (n)p43 glycosylation upon host protection. Here we show that vaccination with a purified native (n)p43 together with adjuvant protected mice against a low dose challenge infection, and protection was dependent upon IL‐4Rα mediated Type 2 immunity. Vaccination with a recombinant (r) p43 was poorly efficacious by comparison to np43 but did significantly stunt the development of adult worms and reduced parasite fecundity. Furthermore, deglycosylation of np43 significantly impaired protection, suggesting that p43 glycans play a role in vaccine induced protection. Finally, adenoviral‐vectored delivery of p43 failed to induce protection to low dose challenge despite the generation of a strong T cell dependent anti‐np43 specific antibody response.

## 2 Methods

### 2.1 Animals

Both 6 and 8 weeks of age male and female mice were used. C57BL/6 mice were purchased from Envigo (C57BL/6JOlaHsd) or Charles River (C57BL6NCrl). AKR/J and C57BL/6IL‐4Rα KO mice [22] and corresponding wild type (WT) littermate controls were bred in‐house at the Biological Services Facility (BSF) at the University of Manchester. Experiments were performed under the regulations of the Home Office Scientific Procedures Act (1986), Licences P043A082 and PP0172300 and were subject to local ethical review by the University of Manchester Animal Welfare and Ethical Review Body (AWERB) and followed ARRIVE 2.0 guidelines. The mice utilised in these experiments were not randomised but cages were randomly assigned to different treatment groups for vaccination experiments.

### 2.2 Antigen Preparation

Native p43, np43, was prepared as previously described [21]. Briefly, culture medium from adult *T. muris* worms were incubated overnight at 4°C with Ni‐NTA agarose (Qiagen, UK) as p43 possesses a natural poly histidine C terminus and readily binds to nickel beads. The next day, after washing the Ni‐NTA agarose beads with 20 and 40 mM Imidazole in 25 mM Tris, 150 mM NaCl pH 7.9, np43 was eluted in 250 mM Imidazole. p43 was further size purified using a Superdex 75 size purification column (GE Amersham). p43 containing fractions in 20 mM Tris, 50 mM NaCl were concentrated, syringe filtered (0.22 μm), aliquoted and stored at −80°C until use. Purity was confirmed by mass spectrometry. PNGase F (New England Biolabs) was used for deglycosylation over a 24‐h period at 37°C as per the manufacturer's instructions for native conditions. A sample of np43 was incubated under identical conditions but lacking PNGase F to generate control (deglycosylated) np43. After incubation, the np43 was isolated from the deglycosylation reaction using Ni‐NTA agarose overnight as previously described and dialysed against 20 mM Tris, 50 mM NaCl buffer pH 7.4. The deglycosylated p43 and control p43 were then dialysed against 20 mM Tris, 50 mM NaCl pH 7.4. Adult *T. muris* ES antigens depleted of p43 are designated ES^−p43^.

### 2.3 Recombinant, Baculovirus‐Derived p43 Production, rp43

This was prepared as described [21]. Briefly, the p43 gene minus signal peptide (aa 18–394) was amplified using a DNA template originally taken from a *T. muris* cDNA library as described previoulsy [21].

### 2.4 Vaccination Protocol

A standard protocol was used throughout to vaccinate mice subcutaneously (s.c.) with 50 μg of either np43, ES^−p43^ or rp43. For control vaccinations both 20 mM Tris, 50 mM NaCl plus Alum (Imject, Thermofisher) and Tris buffer alone were used. For Alum formulations, 50 μg protein or Tris buffer was mixed at a 1:1 volume with Thermo Scientific Imject Alum Adjuvant for at least 30 min. Mice were injected s.c. twice, 28 and 14 days prior to challenge (D‐28 and D‐14). On day 0, mice were infected with 20 *T. muris* eggs by oral gavage for low dose or 200 eggs from a stirring suspension for high dose infections. Mice were usually culled 35–42 days later for quantification of adult worm burdens and antibody analysis.

### 2.5 Non‐Replicating Adenoviral p43 Vaccination

The nucleotide sequence for p43 was obtained from the GenBank sequence database (TMURIS_00086910) and was codon‐optimised for expression in mammalian cells (Figure S1). The recombinant human serotype 5 adenoviral vector HuAd5 p43 was generated using a Gateway‐compatible entry vector using a CMV promoter and a tPA signal sequence as described previously [23]. C57BL/6 mice were vaccinated at day −42 intramuscularly with ≤ 10^9^ infectious units HuAd5 p43 or empty vector HuAd5 as a control. Mice were anaesthetised, shaved and injected on the hind leg. At day −28, a group of mice were vaccinated s.c. with ES^−p43^ as a positive control or a negative control (Alum plus buffer) as in the standard protocol. On day 0, mice were infected with a low dose of *T. muris* eggs and assessed for worm burdens and *T. muris* specific antibodies on day 35 post infection.

### 2.6 Analysis of T Cell Responses

Mesenteric lymph node cells (MLNC) were harvested to measure antigen‐specific cytokine secretion on day 21 post infection (p.i) as described previously [21]. Cytokine analysis was carried out as previously described [21] using Cytometric Bead Array (BD Biosciences) and analysed on a MACS Quant (Miltenyi). The following cytokines were measured in response to the antigen stimulation: IL‐4, IL‐9, IL‐10, IL‐13, IFN‐g & IL‐17. The data was analysed using FCAP Array Vs 3.0 (BD Biosciences).

### 2.7 Antigen Specific Antibody ELISAs

Pre‐challenge infection serum was collected from the tail vein and post challenge serum collected by cardiac puncture following euthanasia. Blood was allowed to clot at room temperature for an hour and then centrifuged at 12,000 g for 10 min. Serum was stored at −20°C until analysed. Antibody ELISAs (IgG1 and IgG2a/c) were carried out as described [21].

### 2.8 Image Analysis of Worms

Experimental worms post infection were fixed using Bles fluid, mounted on microscope slides, and imaged using a Leica snapshot dissecting microscope (Bioimaging Core Facility at the University of Manchester). Worm length was measured using Image J.

### 2.9 Faecal Egg Counts

Two faecal pellets were taken from individual mice and weighed. Egg counts were assessed using water to resuspend the pellets. An equal volume of saturated NaCl was added to the suspension and eggs were counted on a McMaster counting slide using a Leica dissecting microscope.

### 2.10 Statistics

All statistical analysis was performed using GraphPad Prism (GraphPad Software). Data distribution was tested for normality using the Shapiro–Wilk test. Statistical tests used are described in the Figure legends. For normally distributed data, Student's *t*‐test was used for comparison between two groups; for three or more group comparisons, one way ANOVA was performed. For non‐normally distributed data, the Mann–Whitney test was used for comparison between two groups; for three or more groups, the Kruskal‐Wallis test was used.

## 3 Results

### 3.1 Immunisation With p43 Protects Against Low Dose Chronic Infection and Generates a Strong Type 2 Immune Response

We have previously shown that np43 can protect against a low dose chronic challenge infection in mice [21]. The mechanism of protection generated by this vaccination regime remains undefined. np43 protected against a *T. muris* chronic challenge infection, leading to a mean reduction of > 90% in adult worms compared to mice which received a control vaccination of alum plus buffer (Figure 1a). Serum from protected mice at day 42 post challenge had higher levels of anti‐*T. muris* ES IgG1 and lower levels of IgG2a/c in comparison to control mice (alum immunised, infected; Figure 1b,c). Antibody responses in the np43 vaccinated mice indicated that protection was associated with high IgG1 and low IgG2a/c (proxies of a Type 2 immune response) against *T. muris* [24]. To explore the cellular immune response to p43, lymph node cells from vaccinated mice were assessed for antigen‐specific cytokine secretion on day 21 post challenge, using *T. muris* ES antigens. Cytokine analyses indicated that vaccination with np43 generated a strong Type 2 response with elevated levels of IL‐4, IL‐9, IL‐10 and IL‐13 (Figure 1d–g) in comparison to control alum immunised infected mice, which showed elevated antigen specific production of IFN‐γ and IL‐17 (Figure 1h,i), characteristic of the Type 1/17 response that underpins chronic *T. muris* infection [18, 25].

**FIGURE 1:**
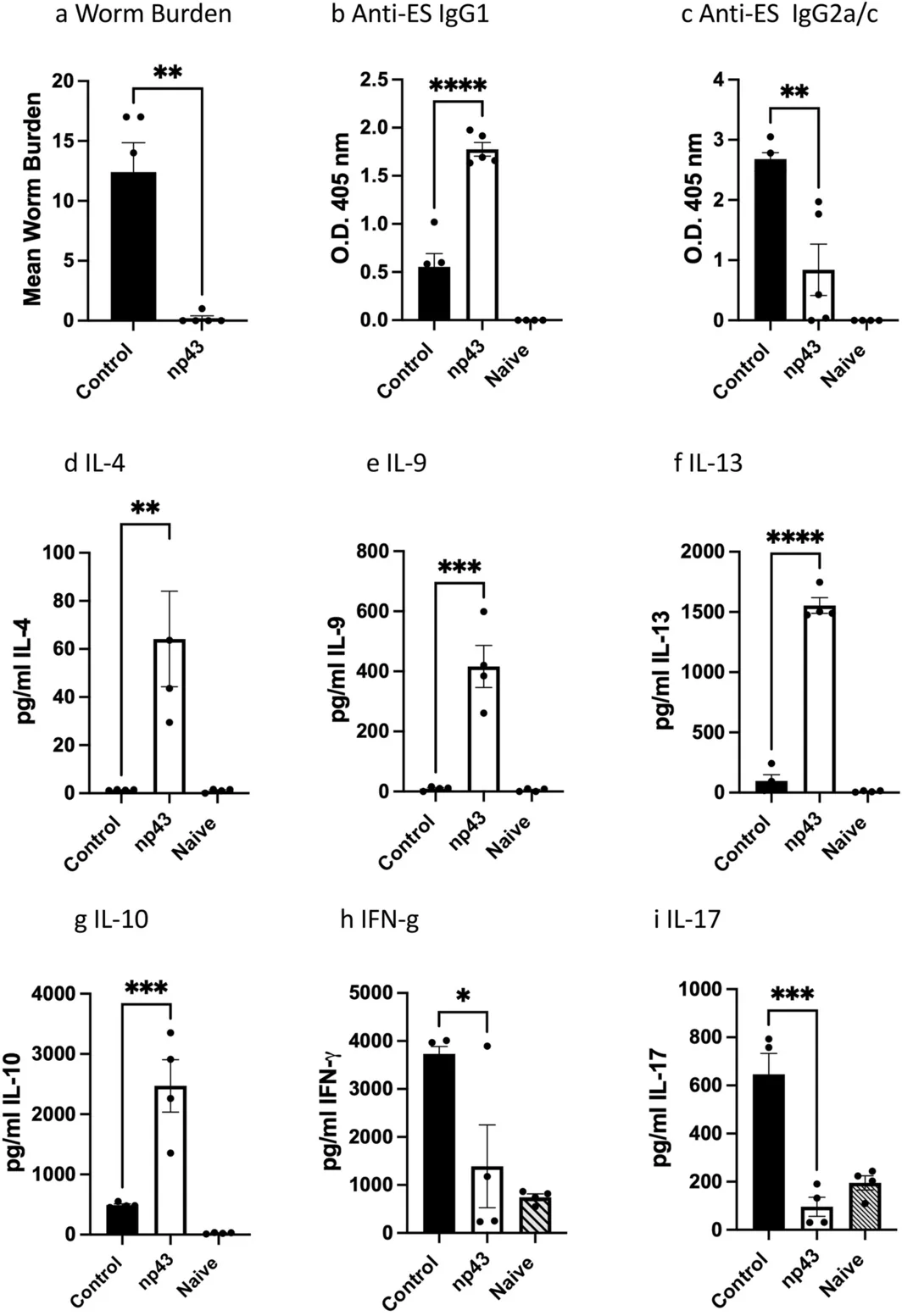
p43 protects against low dose chronic infection and generates a strong Type 2 immune response. (a) Worm Burdens from C57BL/6 mice vaccinated with two doses of np43. Control mice were vaccinated with Alum and buffer. The mice were infected by oral gavage with 20 *T. muris* eggs on day 0. Worm burdens were assessed at day 42 p.i. (b and c) IgG1 and IgG2a/c antibody analysis from vaccinated mice at day 42 p.i. measured by ELISA against ES antigens. (d–i) Cytokines were analysed from supernatants of MLNC re stimulated with ES antigens at day 21 p.i.(d–i) shows levels of IL‐4, IL‐9, IL‐13, IL‐10, IFN‐γ and IL‐17. Analysis was carried out by CBA and analysed using Fcap Array 3.0 software. Naïve = non vaccinated, not infected. Data shown with mean ± SEM *n* = 4–6 mice. * indicates *p* value ≤ 0.05, ** indicates *p* value ≤ 0.01, *** indicates *p* value ≤ 0.001, **** indicates *p* value ≤ 0.0001.

### 3.2 p43 Vaccination in Mice Lacking Th2 Responses Fails to Protect Against a Low Dose Challenge Infection

To assess the importance of Type 2 immune response in vaccine induced protection against *T. muris*, mice which lacked the capacity to signal through the IL‐4Rα and were unable to respond to signals from the canonical Type 2 cytokines IL‐4 and IL‐13 were vaccinated and challenged with a low dose infection. The high level of protection seen to challenge in immune competent wild type C57BL/6 mice was completely abrogated in IL‐4Rα KO mice (Figure 2a). Both anti‐p43 IgG1 and IgG2a/c were detected in p43 vaccinated IL‐4Rα KO mice pre‐infection (Figure 2b,c). As previously observed, there was no anti‐p43 antibody response seen in control alum immunised infected mice [21].

**FIGURE 2:**
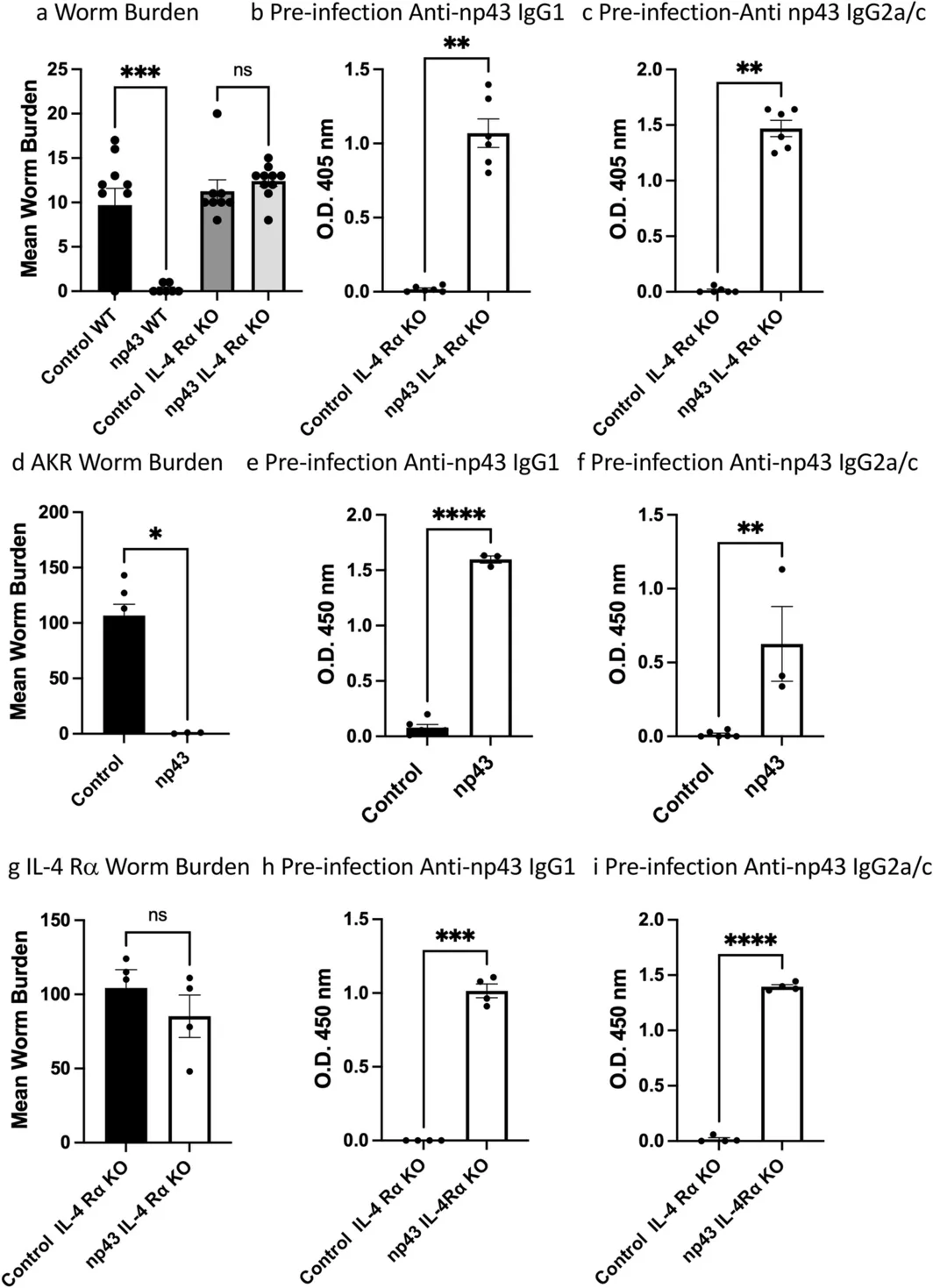
Vaccination of mice lacking Th2 responses does not protect against infectious challenge. IL4 RαKO mice and wild type littermates (WT) were vaccinated twice with p43 (np43 IL‐4Rα KO) or Alum as a control vaccination (control IL‐4Rα KO). Mice were infected by oral gavage with 20 *T. muris* eggs on day 0. (a) Worm burdens were assessed on day 35 p.i. Anti‐np43 IgG1 (b) and IgG2a/c (c) were analysed by ELISA using a 1/80 dilution of serum. AKR mice were vaccinated s.c. with np43 (np43) or a control vaccination (control) and infected on day 0 with 200 *T. muris* eggs. (d) Worm burdens were assessed on day 35 post infection. (e and f) On day −1 a sample of blood was removed from the tail vein and serum prepared for anti‐np43 IgG1 and IgG2a/c responses. ELISA analysed at serum dilution of 1/80. (g–i) IL‐4 RαKO mice were vaccinated with p43 (np43 IL‐4Rα KO) or a control vaccination (control IL‐4Rα KO). Mice were infected on day 0 with 200 *T. muris* eggs. Worm burdens were assessed on day 14 p.i., (g) and anti‐np43 IgG1 and IgG2a/c ELISA responses were assessed using a 1/80 dilution of serum, (h and i). Data shown with mean ± SEM *n* = 4–9 mice. Three experiments *n* = 3–5. * indicates *p* value ≤ 0.05, ** indicates *p* value ≤ 0.01, *** indicates *p* value ≤ 0.001, **** indicates *p* value ≤ 0.0001, ns = non‐significant.

Most vaccination studies with *T. muris* have used a high dose challenge infection to measure protective immunity. AKR/J mice are one of the few inbred strains of mouse naturally susceptible to a high dose *T. muris* infection as they do not expel their worm burden. The failure to expel is due to the generation of a Type 1 (IFN‐γ) response and suppression of Type 2 immunity [26]. We show here that AKR/J mice vaccinated with np43 could be successfully protected against a high dose challenge infection (Figure 2d). Pre‐challenge antibody analysis revealed significantly elevated anti‐np43 IgG1(Figure 2e) and IgG2a/c (2f) antibody responses. Again, alum only‐immunised AKR/J mice had no anti‐np43 antibody response following subsequent infection. Also, vaccination of IL‐4RαKO mice with np43 failed to protect against a high dose challenge infection (Figure 2g), despite the presence of anti‐np43 IgG1 and IgG2a/c antibody prior to challenge, (Figure 2h,i). No np43 specific antibody was observed in control alum immunised infected IL‐4RαKO mice.

### 3.3 Vaccination With HuAd5 p43 Generates Strong Antibody Responses Against p43 but Does Not Protect Against Challenge Infection

Vaccination using replication‐deficient adenoviruses is successful in protection against several infectious diseases such as Ebola [27, 28] and group B meningococcal disease [29].

An adenoviral vaccine encoding p43, HuAd5 p43, was generated. Mice were vaccinated intramuscularly (i.m.) 6 weeks prior to a low dose challenge infection. An empty adenovirus vector, HuAd5 was used as a negative control. Vaccination with HuAd5 p43 failed to protect mice against a chronic low dose challenge infection, as no reduction in worm burden was observed (Figure 3a) compared to control vaccinations (HuAd5). Pre‐infection sera from HuAd5 p43 vaccinated mice indicated that the vaccine induced significant levels of anti‐np43 IgG1 and anti‐np43 IgG2a/c (Figure 3b,c) while no antibody responses to np43 were seen in the control vaccinated groups. Following infection, all groups had significantly elevated anti‐ES^−p43^ IgG1 and 2a/c responses to non p43 ES antigens (Figure 3d–g) with no differences in antibody levels between any of the infected groups.

**FIGURE 3:**
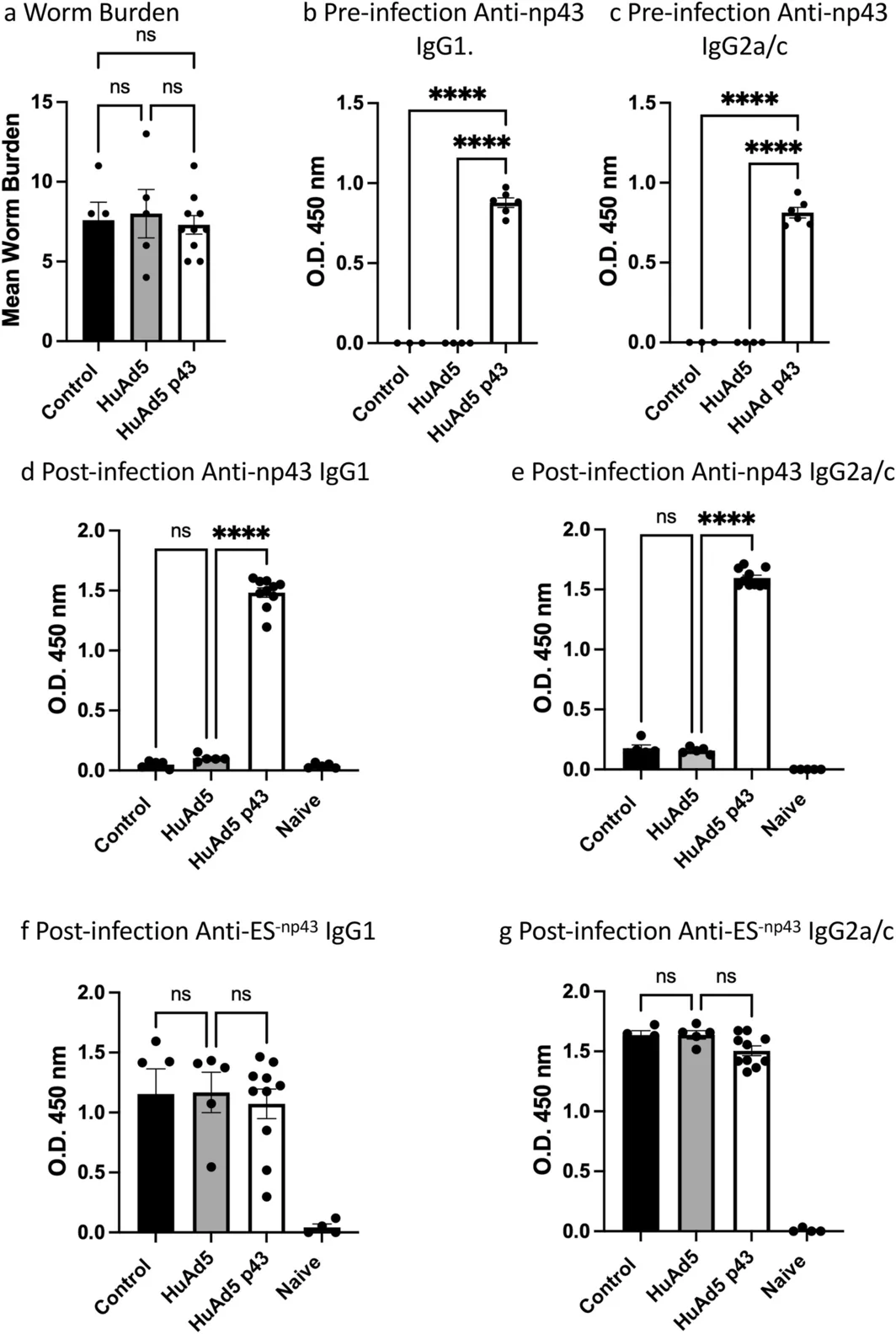
Immunisation with HuAd5 p43 induces strong antibody responses against p43 but does not protect against infection. Mice received ≤ 10^9^ infectious units of HuAd5 p43 or empty HuAd5 (control) i.m. 6 weeks prior to challenge. As a positive control a group of mice were vaccinated s.c. with 50 μg with ES‐^p43^ twice, at day 28 prior to challenge and boosted ‐day 14 prior to challenge. On day −1 a sample of blood was removed from the tail vein and serum prepared for antibody analysis. On day 0 all mice were given 20 *T. muris* eggs by oral gavage. On day 35 p.i. mice were culled. (a) Worm burdens were assessed. (b and c). Pre‐infection anti‐np43 IgG1 (b) and IgG2a/c (b) were performed. Post infection anti‐np43 IgG1 (d) and IgG2a/c (e) are shown and anti‐ES^−p43^ IgG1 (f) and IgG2a/c (g) ELISA. Pre‐infection ELISAs were performed using a serum dilution of 1/80. Data shown with mean ± SEM *n* = 5–10 mice, **** indicates *p* value ≤ 0.0001, ns = non‐significant.

### 3.4 Vaccination With Recombinant p43, rp43, Results in Stunted Worms and Reduced Fecundity of Adult Worms

Production of np43 requires large numbers of live adult parasites to generate sufficient antigen. For any future development of p43 orthologues of human or animal whipworms, this is not a feasible approach, and an effective recombinant antigen would be advantageous. In order to assess the efficacy of *recombinant* p43 for vaccination against mouse whipworm, baculovirus derived recombinant (r)p43 was used to vaccinate mice, and to assess the protective capacity against a low dose challenge infection using the same adjuvant and two dose regime that was successfully used with np43. Vaccination with rp43 failed to consistently generate a significant reduction in worm burden (Figure 4a). Vaccination with rp43 did however result in significantly stunted worms in comparison to control alum immunised mice and reduced the overall wet biomass of recovered worms/treatment group when compared to the infect alone group (Figure 4b,c). Mice vaccinated with np43 and rp43 had significantly elevated anti‐np43 IgG1 (Figure 4d) but not IgG2a/c (Figure 4e) titres, following challenge, while control alum immunised mice had no anti‐np43 antibody responses. Mice vaccinated with np43 and rp43 had anti‐whole ES IgG1 and IgG2a/c following challenge infection as did control alum immunised mice (Figure 4f,g). Importantly, the data demonstrate that mice vaccinated with rp43 generate a strong antibody response to the np43 molecule. The anti‐ES IgG1 results show a significantly higher level of IgG1 in the np43 vaccinated mice compared to rp43 vaccinated mice (Figure 4f). All infected groups showed equivalent elevated levels of anti‐ES IgG2a/c (Figure 4g).

**FIGURE 4:**
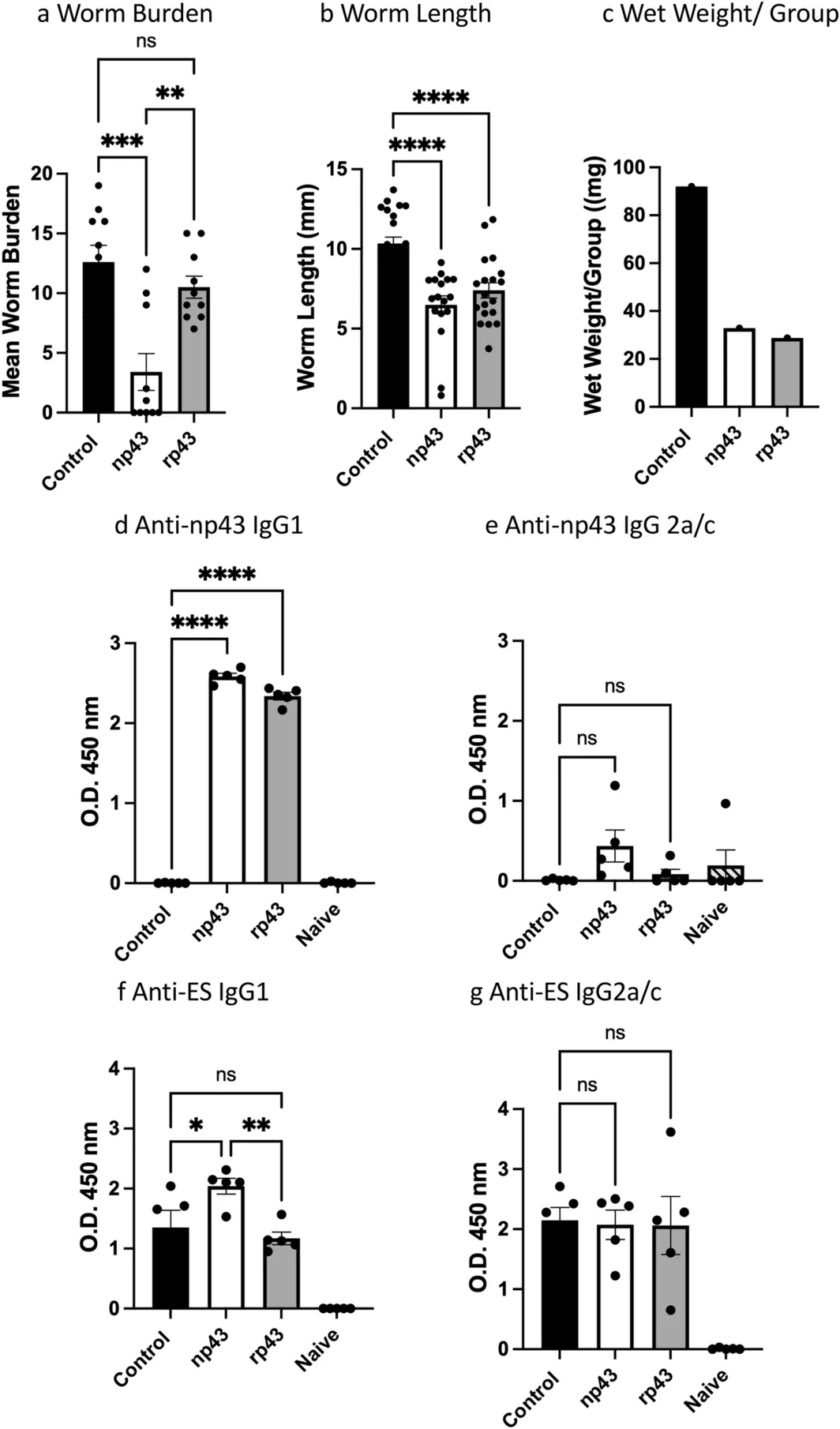
Immunisation with recombinant p43, rp43, results in stunted worms and reduced fecundity of adult worms post challenge. np43 or rp43 together with Alum adjuvant were used to immunise mice twice. Mice were challenged and at day 35 post challenge, worm burdens were assessed; (a) worms were fixed and worm lengths measured; (b). Wet weight of worms per experimental group were assessed; (c). (d) shows anti‐np43 IgG1 and (e) shows anti‐np43 IgG2a/c responses at a serum dilution of 1/80. (f, g) show anti‐ES IgG1 and IgG2a/c. Data shown with mean ± SEM *n* = 5–10 mice. ** indicates *p* value ≤ 0.01, *** indicates *p* value ≤ 0.001, **** indicates *p* value ≤ 0.0001. ns = non‐significant.

### 3.5 PNGase F Treatment of Native p43 Partially Abrogates Protection to a Chronic Challenge Infection

p43 has two potential glycosylation sites [21]. As vaccination with np43 induced superior protection compared with rp43, we examined whether glycosylation affected the np43 vaccination‐induced protection. Mice were vaccinated with de‐glycosylated np43 and glycosylated np43. PNGase F treatment abrogated the protection seen with np43 as shown by significantly reduced intestinal worm burdens (Figure 5a) and reduced faecal egg counts (Figure 5b). ELISA on Pre‐infection serum showed that vaccinated mice had mounted anti‐np43 IgG1 and IgG2a/c responses (Figure 5c,d), with the glycosylated vaccine inducing a significantly higher level of anti‐np43 IgG1 and a trend towards a lower level of anti‐np43 IgG2a/c. Together these results suggest that glycosylation is important for effective vaccination by p43.

**FIGURE 5:**
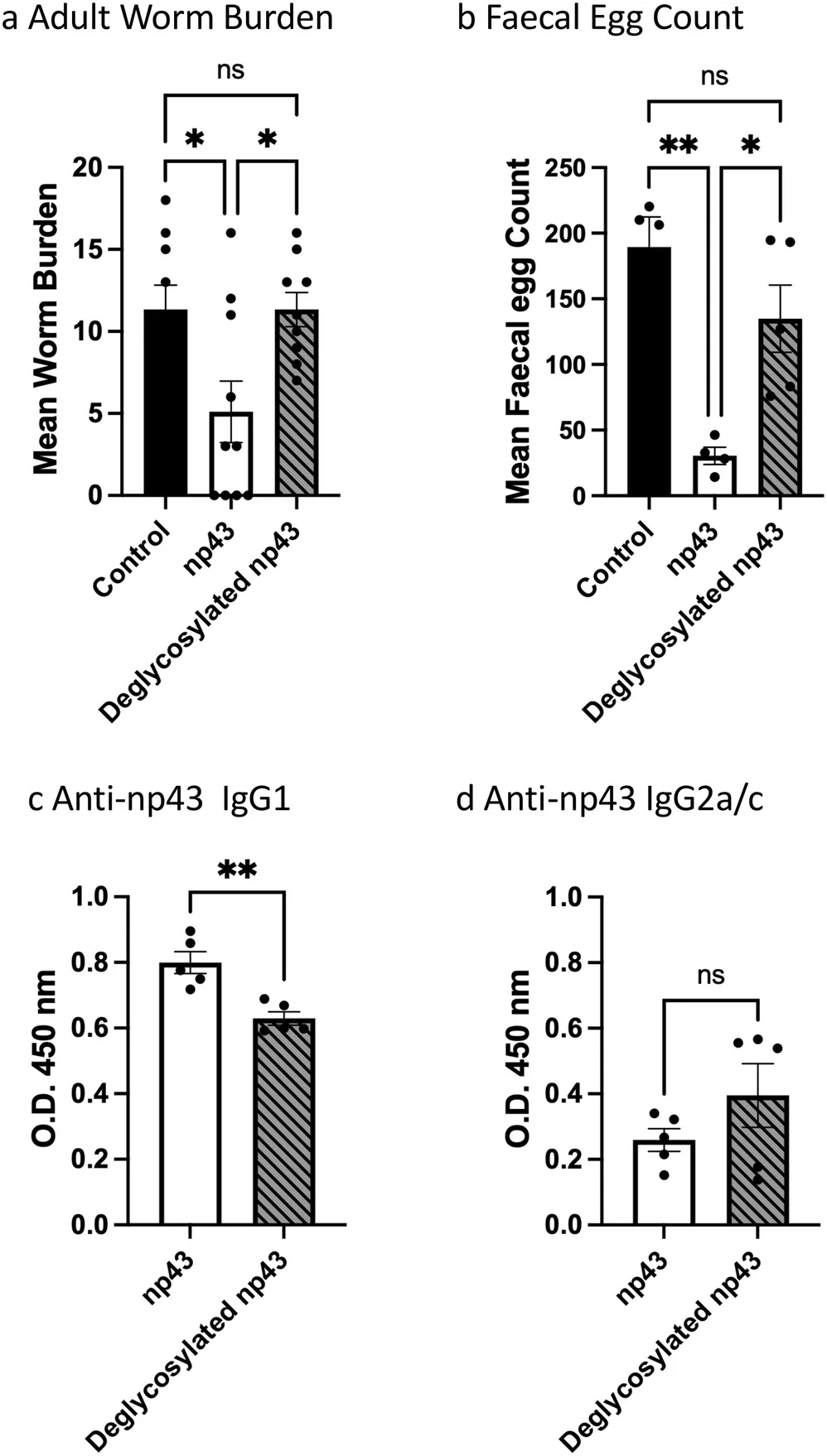
PNGase F treatment of native p43 partially abrogates the vaccine‐induced protection against a chronic challenge infection. Deglycosylation of np43 was carried out and p43 was purified as previously described. Control np43 was treated in the same way without PNGase F added to the reaction mixture. A total of 50 μg of deglycosylated or control np43 formulated in Alum was used to vaccinate mice twice at 2‐week intervals as described above, followed by challenge 2 weeks later on day 0. At day 35 post challenge, worm burdens were assessed (a) and faecal egg counts assessed (b). Pre infection anti‐np43 IgG1 (c) and IgG2a/c (d) were assessed using a serum dilution of 1/100. Data shown with mean ± SEM *n* = 5–10 mice. Pooled data from 2 to 3 experiments. * indicates *p* value ≤ 0.05, ** indicates *p* value ≤ 0.01. ns = non‐significant.

## 4 Discussion

Here we confirm and extend our earlier observation [21] that a single *T. muris* antigen, p43, the poly‐cysteine and histidine‐tailed protein of *T. muris* induces strong protection to a challenge infection following vaccination when administered with alum adjuvant.

Whilst the majority of experimental *T. muris* vaccination studies have assessed protection by the accelerated expulsion of a high dose challenge infection [15, 16, 17] which would normally be expelled via a Type 2 response, we have chosen here to predominately assess protection against chronic infection using a low dose challenge. We believe this represents a more natural parasite exposure level and tests the capacity of the vaccine to protect against the host response which leads to chronic patent infection, that is characterised by a Type 1 response which is antagonistic to the protective Type 2 response [19]. Vaccination with np43 with alum induced almost complete protection to a low dose challenge infection. This was accompanied by a significantly elevated level of parasite specific IgG1 antibody response and significantly reduced IgG2a/c response compared to control alum alone immunised infected mice. Recall T cell responses in MLNC following challenge infection showed significantly elevated production of IL‐4, IL‐9, IL‐13 and IL‐10 and significantly decreased levels of IFN‐γ and IL‐17 in np43 vaccinated mice compared to controls. This indicated that vaccine induced resistance to p43 was associated with a strong Type2 response and diminished Type 1 response.

To test the functional importance of Type 2 immune responses in np43 vaccine induced protection, vaccinations were performed in IL‐4Rα KO mice. np43 vaccination of mice lacking IL‐4Rα was unable to protect against either a low or high dose challenge infection despite elevated levels of np43 specific IgG1 and IgG2a/c being present before challenge. These observations indicate that IL‐4/IL‐13 signalling is critical to the mechanisms of vaccine induced protection. Vaccination of immunocompetent AKR mice which are naturally susceptible to a high dose *T. muris* infection were completely protected by vaccination with np43 against a high dose challenge and again accompanied by high levels of np43 specific antibody prior to challenge. Interestingly Dixon et al. [15] carried out vaccinations of AKR mice with total adult worm ES with Freund's complete adjuvant using a shorter vaccination protocol and showed similar levels of protection and resistance was also associated with the generation of a Type 2 immune response. Taken together, the data suggest that the efficacy of np43 vaccination requires the generation of a protective type 2 response operating through the IL‐4Rα.

Vaccination using antigen expressed in adenoviral vectors has been shown to generate high titres of protective antibodies against a number of infectious diseases [27, 28, 29]. Vaccination with a HuAd5 p43 adenoviral vaccine failed to protect against a low dose challenge *T. muris* infection. High levels of anti‐np43 IgG1 and IgG2a/c antibody were present prior to and post challenge, although we have no data on the conformation of the p43 molecule when expressed in HuAd5 and how this compares to the structural conformation of np43. Thus, reduced efficacy of vaccination could be related to altered conformation of p43 expressed in HuAd5. The data did show, however, that vaccination with HuAd5 p43 generated antibodies that clearly cross react with np43. Moreover, we did not examine the T cell response to HuAd5 p43 vaccination, although we can infer T cell activation in the response induced by HuAd5 p43 vaccination by the production of antigen specific antibody isotype class switching.

The mechanisms of vaccine induced protective immunity to *T. muris* remain to be defined for any antigen preparation. Certainly, infection induced protective Type 2 immunity to *T. muris* and worm expulsion does not require parasite specific antibody [30]. Effector mechanisms responsible for clearing *T. muris* infection are mediated through IL‐13 driven control [31] of increased intestinal epithelial turnover [32], goblet cell hyperplasia and mucus production [33] and IL‐9 mediated intestinal muscle contraction [34].

Previous work vaccinating mice with a mixture of *T. muris* ES antigens without np43 protein did not support a role for antibody in host protection [13] although presently we cannot rule out a role for antibody‐mediated protection to p43.

Recombinant vaccine antigens against a number of soil transmitted helminths have been trialled both in experimental animals and humans with varied outcomes in terms of protective efficacy. For *Trichuris* several candidates have been investigated experimentally including whey acidic protein (WAP) domain‐containing proteins and cysteine‐rich secretory protein/antigen 5/pathogenesis related −1 (CAP) proteins. Briggs et al. [16] used *T. muris* whey acidic protein (rTm‐WAP49) and *T*. *muris* WAP fragment fusion protein (rTm‐WAP‐F8 + NAGST‐1) together with montanide 720 adjuvant given in three doses to AKR/J mice and showed significant reductions in worm burdens (between 33% and 48%) assessed on day 15 post high dose challenge. Protection was associated with elevated Type 2 responses and a positive parasite specific IgG1/IgG2a/c ratio. In the present study recombinant *T. muris* p43 together with alum adjuvant was used to vaccinate mice given over two doses and challenged with a low dose infection. Consistently, vaccination with rp43 did not significantly reduce worm burdens unlike np43. However, vaccination with rp43 did show evidence of reduced adult worm length, depressed female worm fecundity and reduced wet worm weight comparable to np43 vaccination.

Many nematodes exhibit distinct N‐glycosylation patterns [35] and it is now recognised that many of the protective antigens from helminths carry glycan modifications that are key to host protection [36, 37]. Moreover, deglycosylation of native helminth antigens can lead to a significant reduction in protective capacity [38]. Deglycosylation of np43 markedly reduced its capacity to protect against low dose challenge infection assessed by worm burdens and faecal egg counts. Prior to challenge anti‐np43 specific IgG1 was reduced compared to mice vaccinated with np43 but anti‐np43 specific IgG2a/c antibody levels showed a trend to be increased in mice vaccinated with rp43 compared to mice vaccinated with np43. Crystallisation studies of p43 indicated two potential glycosylation sites [21]. The recent description of the glycosylation of native *T. suis* proteins indicate that they are characterised by varied tri‐/tetra antennary structures carrying PC modified LDN and LDNF motifs [35]. In the present study, rp43 was generated in baculovirus which utilises distinct glycosylation pathways compared to mammals and nematodes [39]. Thus, differences in glycosylation could underlie the reduction in immunogenicity between native and recombinant p43. Indeed, the glycosylation of the HuAd5 p43 vaccine may also impact on its protective capacity.

It is notable that choice of expression system impacts on helminth vaccine efficacy as has been shown for several recombinant intestinal nematode vaccines [40, 41]. Whilst the use of different vaccination regimes in combination with different adjuvants would be one useful avenue to follow to increase the efficacy of rp43, another would be the selection of a recombinant expression system that can be engineered to glycosylate protein antigens similar to parasitic nematodes (e.g., *Nicotiana benthamiana* [42]).

A consistent observation of all the studies presented here is the lack of immune recognition of np43 by the host immune system during *Trichuris* infection (low or high dose) as evidenced by lack of an antibody response despite clear evidence of secretion of p43 by the parasite during infection [21]. It is clear, however, that np43 is potently immunogenic when delivered parenterally with adjuvant and readily generates a strong immune response which is host protective. The reasons for this remain to be defined but may be related to the high affinity of p43 for extracellular matrix molecules such as heparan sulphate found in the parasite niche during infection [21, 43] which may curb/prevent effective access to antigen presenting cells and subsequent antigenic processing of np43 within the epithelial niche.

Experimental studies have shown that mice that have experienced a patent chronic *Trichuris* infection via the induction of a non‐protective Type 1 response are completely susceptible to a subsequent challenge infection even if the initial infection is removed by anthelmintic and regardless of the level of the challenge infection [20]. This contrasts with the outcome seen in animals given a high dose infection which is cleared by a Type 2 response and subsequently challenged with either high or low dose infection. In this case parasites are cleared by anamnestic Type 2 immunity [20, 44]. Thus, animals appear to retain immunological memory associated with the type of immune response they generated upon first exposure to the parasite. More importantly, animals that have experienced a chronic infection are refractory to subsequent vaccination with ES even if the infection has been cleared by drug treatment [45] suggesting that induction of Type 1 immunity negatively influences the subsequent protective response to vaccination. This is likely driven by Type 1 immune responses to non‐p43 antigens during infection which are activated upon subsequent challenge. As the host is not primed to p43 during infection, vaccinating with p43 may be able to avoid (or overcome) the responses promoting susceptibility. Importantly p43 has orthologues in all *Trichuris spp* with known genomes including *T. trichiura* and the recently described *T. incognita* , which infect humans [9, 10]. The data presented here provide a new and effective *Trichuris* vaccine candidate worthy of consideration in the development of integrated control strategies for whipworm infection in the future.

## Author Contributions

A.J.B. performed experimental work, data analysis and wrote the manuscript. K.S.H., L.C., S.T., C.D. and L.B. performed experimental work. R.K.G. provided overall supervision, conceptual guidance, and critical manuscript revision. D.J.T. and C.S.R. critically reviewed the manuscript. All authors contributed to data interpretation, reviewed the manuscript, and approved the final version.

## Funding

This work was supported by Wellcome Trust, Z10661/Z/18/Z, 088785/Z/09/Z.

## Ethics Statement

Experiments were performed under the regulations of the Home Office Scientific Procedures Act (1986), (Licences P043A082 and PP0172300) and were subject to local ethical review by the University of Manchester Animal Welfare and Ethical Review Body (AWERB) and followed ARRIVE 2.0 guidelines.

## Supporting information


**Figure S1:** p43 Nucleotide sequence optimised for production of HuAd‐p43 Adenoviral vaccine.

## Data Availability

Data sharing not applicable to this article as no datasets were generated or analyzed during the current study.
