# Supplementary material for: Vaccination With Trichuris muris Poly‐Cysteine and Histidine‐Tailed Protein p43 Protects Against Chronic Infection
Source: Parasite Immunol. 2026 Sep 30;48(10):e70107. doi: 10.1111/pim.70107 (PMC13626742; doi:10.1111/pim.70107)
Supplement: Supplementary file 1 [file PIM-48-e70107-s001.docx]

**Supplementary Figure**

Figure S1. p43 Nucleotide sequence optimised for production of HuAd-p43 Adenoviral vaccine.

AGAGGATCGAAGCTTGTGGCCATGCTGGTGCTGTTCTTCCCTCTGCTGCTGACCGTGGGACTGTCTACAGCCGGACACGTGAAGTGCCCCGATTTCGGCGATTGGAAGCCCTGGACCGACTGCCTGTGGTATCCTCCTCAGCACATGTACAGCAAGCTGAGCCACGCCTGTGGAATGCACGCCCACAGAAATCTGACCGGCGTGATGGATCTGCCCCACGGCCACAAAACACCTCCACCATGTGGCCACTGCAGCTTCAAGTTCCGGTGCAGAAGAAGGCCCAACACCGAGGGCTGTTACCCTCTGGATGGCGAGGTGGAAGTGTGCCACGACCACAGCGATATCTGCACCCTGCCTAAGCTGCCTCACCTCGGCTGTGGCTACGCCTTCATCAACGAGAAGCTGAAGCAGTGCTTTACCCGGCCTGACACACCTAGCTATGTGCGGCTGGGCTACAGAAAGATGTTCGAGAGCATCCCCAAGAAGCACTGCATCGAGAAGGACGGCATGTGCAAGTGCTGCTGCGGCGACTACGAGCCTAATGAGAGCGGCACCGAGTGCATCAAGCCTCCAGCTCACGATTGCCCTGCCTACGGACCTCCATCTGAGTGGAGCGAGTGTCTGTGGTTCCCTCTGAAGAACATCGTGTCCCACGTGTACGACCACTGCCACGTGCACAAAGAGCCTGACGGATACGAGCCTCACAGCGTGGCCCCTAGAGACAAGAAGGATGGCTGCTTCCCACTGAAGCTGGGCAAGAAGTCTTGCGGCAAGGACGACTGTCCTACCTGTGGCGACATCTGTACCCTGGACAAGATCAACGGCAGCTGCGCCTTTCCTAGAGTGATGAAGGAAAAGATCTGGGACGACTTCACCGCCACCAGCAAAGAAAAGCACATGCCCCACTGGAAGCGCGACGGCTATGCCAAGATGCTGATGCAGCTGCCCTACAGCAACTGCAAAGAAGTGGGCGACAAGTGCAAGTGTTGTTGTCACCCCTACGAGCCCAACAAGGATGGCACAGCCTGCGTGGTCAAAGAATACTGCAAGCGGGTGCACGAGCTGCACCACCACGATCATCAGGACACGGCGAGGAACACCACAAGAGCAGCAGCAGCGAGAGCAAAGAGCACCACCATCACTGAGCGGCCGCGGGGTCT
